# Methodological concerns and data discrepancies in the indirect treatment comparison of belimumab and anifrolumab

**DOI:** 10.1007/s00393-026-01838-w

**Published:** 2026-06-23

**Authors:** Kai Wai Lee, Becky Hooper, Jason Steenkamp, Ian N Bruce

**Affiliations:** 1 GMAP, BioPharmaceuticals, AstraZeneca, Cambridge, UK; 2 EVERSANA, Victoria, British Columbia Canada; 3 Manchester Academic Health Science Centre, The University of Manchester, Manchester, UK

## Original publication

Lee YH, Song GG (2026) Indirect comparative efficacy and safety of belimumab vs. anifrolumab in systemic lupus erythematosus and lupus nephritis: a meta-analysis of randomized trials. Z Rheumatol 85:298–306. https://doi.org/10.1007/s00393-025-01760-7.

Dear Editor,

we are writing to address several significant methodological and scientific concerns in the recent article “Indirect comparative efficacy and safety of belimumab vs. anifrolumab in systemic lupus erythematosus and lupus nephritis: a meta-analysis of randomized trials” by Lee and Song.

The authors acknowledged as a limitation of their study that the analysis is based solely on published aggregated results, which may introduce bias and limit the ability to adjust for inter-study variability. This contrasts with existing published indirect treatment comparisons (ITCs) from the same trials that compared individual patient data (IPD) from anifrolumab to aggregated results from belimumab and vice versa. These published population-adjusted ITCs have demonstrated comparable or favorable results for anifrolumab over belimumab in terms of the Systemic Lupus Erythematosus Responder Index 4 (SRI-4). Given this limitation and the existing publications which employ more robust methodology, it is not clear what this study adds to the body of scientific knowledge beyond the already published ITCs.

We also have concerns related to the data used in the analysis. We have been unable to verify the data analyzed for some outcomes, despite the data availability statement which notes that all data analyzed during the study are included in the article. Anti-double-stranded DNA (anti-dsDNA) normalization and complement 3 (C3)/complement 4 (C4) normalization were reported as the median change from baseline in the primary publications for TULIP‑1 and TULIP‑2 but were assessed as dichotomous outcomes in this article. Only TULIP‑1 SRI‑4 efficacy results were used in the primary analysis, when TULIP‑2 was the primary pivotal trial. For TULIP‑1 SRI‑4, the authors used the prespecified rules in their analysis, whereas the amended rules for restricted medications were accepted by regulatory agencies such as the U.S. Food and Drug Administration (FDA) and the European Medicines Agency (EMA). These issues mean that the paper does not consider the context upon which the analysis is based, which again limits any conclusions which may be drawn.

The lupus nephritis (LN) efficacy indirect treatment comparison was based on a significant disparity in data maturity. It is methodologically inconsistent to draw definitive conclusions by comparing an exploratory, dose-finding phase II trial (anifrolumab) with a pivotal phase III trial (belimumab). Given that the phase III IRIS trial for anifrolumab is ongoing and uses a different dosing regimen, any indirect treatment comparison at this stage is premature and potentially misleading.

There are also reporting and methodological errors. The labelling in figures 2 and 3 indicates that values to the *left *of the line favor belimumab, yet the opposite is the case—for example, the odds ratio (OR) reported in the text for achieving steroid reductions clearly favors anifrolumab, contradicting the labelling in Fig. 2. An exception is the severe flare rate, where belimumab had a higher severe flare rate than anifrolumab (OR 1.25, 95% confidence interval [CI]: 0.73–2.14), thus consistent with the labelling of the figure. In the text and Table 3, the authors mentioned that comparable results for anifrolumab were not available for the risks of renal event and death in LN. This is contradicted in Fig. 3 by showing nominal significance in favor of anifrolumab. Conclusions for safety outcomes are even less robust, as these are based on a simple qualitative comparison of event rates in the single active arms without accounting for the placebo arms of the trials.

We also note several scientific inaccuracies in the article. For example, the authors state that B‑lymphocyte stimulator (BLyS) leads to decreased auto-antibody production. In actual fact, BlyS promotes B‑cell survival and leads to increased auto-antibody production. The authors also incorrectly identify BLyS as the first biologic approved for SLE, yet belimumab is actually an anti-BLyS biologic. They also failed to acknowledge the evolving treatment landscape, where the European Alliance of Associations for Rheumatology (EULAR) and the American College of Rheumatology (ACR) recently published clinical guidelines recommending treatment to the target definition of remission in SLE (DORIS) and lupus low disease activity state (LLDAS), and instead emphasized the importance of SRI‑4.

These methodological and scientific discrepancies significantly limit the validity of the results, and it is unclear what this study contributes to the scientific body of knowledge beyond the already published ITCs. The limitations we have highlighted need to be borne in mind when reviewing the study, and the conclusions interpreted with caution.

## Data Availability

All data supporting the findings of this work are included in the article.

## References

[1] Lee YH, Song GG (2025) Indirect comparative efficacy and safety of belimumab vs. anifrolumab in systemic lupus erythematosus and lupus nephritis: A meta-analysis of randomized trials. Z Rheumatol. 10.1007/s00393-025-01760-7 41342907

[2] Bruce IN, Golam S, Steenkamp J, Wang P, Worthington E, Desta B, Psachoulia K, Erhardt W, Tummala R (2022) Indirect treatment comparison of anifrolumab efficacy versus belimumab in adults with systemic lupus erythematosus. Journal of Comparative Effectiveness Research 11(10):765–777. 10.2217/cer-2022-0040 35546484

[3] Neupane B, Shukla P, Slim M, Martin A, Petri M, Bertsias GK, Kim AHJ, Fanouriakis A, Levy RA, Chauhan D, Ballew N (2023) Belimumab versus anifrolumab in adults with systemic lupus erythematosus: An indirect comparison of clinical response at 52 weeks. Lupus Science & Medicine 10(1):e000907. 10.1136/lupus-2023-000907 PMC10186457 37147022

[4] Fanouriakis A, Kostopoulou M, Anders HJ, Andersen J, Aringer M, Beresford MW, Doria A, Frangou E, Furie R, Gladman DD, Houssiau F, Karras A, Kouloumas M, Madenidou AV, Malvar A, Marinaki S, Mok CC, Moroni G, Parodis I, Rednic S, Sieiro Santos C, Scirè CA, Smolen JS, Tamirou F, Tanaka Y, Teng YKO, Welin E, Bertsias G, Boumpas DT (2026) EULAR recommendations for the management of systemic lupus erythematosus with kidney involvement: 2025 update. Ann Rheum Dis 85(1):75–90. 10.1016/j.ard.2025.09.007 41107121

[5] Sammaritano LR, Askanase A, Bermas BL, Dall’Era M, Duarte-García A, Hiraki LT, Son MBF, Werth VP, Aranow C, Barnado A, Broder A, Brunner HI, Chong BF, Chowdhary VR, Hersh AO, Izmirly PM, Jules M, Kalunian K, Kamen D, Rubinstein TB, Smith BJ, Smith NM, Thomas A, Timlin H, Wallace DJ, Azzam M, Bartels CM, Cunha JS, DeQuattro K, Fava A, Figueroa-Parra G, Garg S, Gomes LLA, Cuéllar-Gutiérrez MC, Iyer P, Johannemann AS, Jorge A, Kasturi S, Kawtharany H, Khawandi J, Legge A, Liang KP, Lockwood MM, Sanchez-Rodriguez A, Turgunbaev M, Williams JN, Turner AS, Mustafa RA (2025) 2025 American College of Rheumatology (ACR) guideline for the treatment of systemic lupus erythematosus. Arthritis Care Res. 10.1002/acr.25690 41182321

[6] Richard A, Furie Eric F, Morand Ian N, Bruce Susan, Manzi Kenneth C, Kalunian Edward M, Vital Theresa, Lawrence Ford Ramesh, Gupta Falk, Hiepe Mittermayer, Santiago Philip Z, Brohawn Anna, Berglind Raj, Tummala (2019) Type I interferon inhibitor anifrolumab in active systemic lupus erythematosus (TULIP-1): a randomised controlled phase 3 trial. The Lancet Rheumatology 1(4):e208–e219. 10.1016/S2665-9913(19)30076-1 38229377

[7] Morand EF, Furie R, Tanaka Y, Bruce IN, Askanase AD, Richez C, Bae S-C, Brohawn PZ, Pineda L, Berglind A, Tummala R (2020) Trial of Anifrolumab in Active Systemic Lupus Erythematosus. N Engl J Med 382:211–221. 10.1056/NEJMoa1912196 31851795

